# Antimicrobial-Resistance of Food-Borne Pathogens

**DOI:** 10.3390/antibiotics10040372

**Published:** 2021-04-01

**Authors:** Pierluigi Aldo Di Ciccio

**Affiliations:** Department of Veterinary Science, University of Turin, Largo Paolo Braccini 2, Grugliasco, 10095 Torino, Italy; pierluigialdo.diciccio@unito.it

The antimicrobial-resistance (AMR) in bacteria represents a major challenge for public health. The misuse and or overuse of antibiotics in livestock is known to have contributed to the emergence of AMR. To date, there is growing concern over the possibility of AMR transmission via the food chain [1]. AMR food-borne pathogens in food-producing animals can spread to humans via consumption of contaminated food or water, and direct contact with animals [2]. Moreover, bacteria in food that is AMR are likely to be more persistent in food processing environments [3]. With regard to this, monitoring of AMR in the food chain is crucial for understanding the diffusion of resistance, providing relevant risk assessment data.

This Special Issue includes full research articles and one review focused on the emergence of AMR in food chain.

A critical problem is the existence of bacteria in a state of latency induced by stressful conditions called “viable but nonculturable (VBNC) cells”. The main aim of the study carried out by Texeira and co-workers was to conduct a thorough analysis of the efficacy of chemical disinfectants by assessing *Escherichia coli* viability in lettuce with flow cytometry. The authors showed that this technique is a powerful tool for rapidly evaluating the effect of disinfection on *Escherichia coli* viability in lettuce. Additionally, the detection of VBNC cells is possible within a few minutes, which avoids their persistence, spread and, consequently, food-borne outbreaks [4].

The genotypic and phenotypic characteristics of biofilm producing and AMR in *Staphylococcus aureus* from milk was the focus of the contribution by Agbortabot Bissong and co-workers. The high frequency of AMR *Staphylococcus aureus* strains in milk intended for human consumption demonstrates the importance of this multi-drug resistant bacteria in terms of public health in the North West Province of South Africa [5]. The occurrence of AMR among *Salmonella* serovars isolated from foods in Italy was investigated by Peruzy and co-workers. A high prevalence of AMR and multi-drug resistant *Salmonella* strains was found [6]. The contribution of Xu and co-workers focused on the AMR properties and patterns of *Salmonella* Rissen isolates from humans, animals, animal-derived products and the environment in China. A high incidence of AMR among *Salmonella* Rissen isolates recovered from various sources was observed [7]. In their contribution, Casagrande-Proietti and co-workers evaluated the *Salmonella* Infantis antimicrobial susceptibility pattern and the prevalence of *Salmonella* Infantis Extended Spectrum β-Lattamase (ESBL) isolated from the broiler food chain and humans in Italy. The authors showed the great diffusion of ESBL multi-drug resistant *Salmonella* Infantis strains [8]. Zhou and coworkers investigated the genotypic and epidemiology characteristics of *Staphylococcus aureus* from healthy pigs, diseased pigs and environment. The multidrug resistance observed among various isolates showed that pigs and pork may have an important role in AMR dissemination [9]. The study of Abass and co-workers focused onthe antibiotics used by farmers and the occurrence of AMR *Escherichia coli* isolates form ready-to-eat meats in Ghana. They showed that the antibiotics used by farmers have a strong link with the resistance pattern of bacteria isolated from animals. With regard to this, ready to eat meats may be a source of AMR *Escherichia coli* isolates [10]. The main aim of the study carried out by Cuccato and co-workers was to evaluate the impact of different prophylactic antimicrobial protocols on the composition and diversity of the gut microbiota in poultry. The authors demonstrated that antimicrobial prophylactic protocols are responsible for gut microbiota compositional alterations.

Anyway, further investigations focusing on the selective pressure applied by antimicrobial treatments on the expression of AMR genes in gut microbiota need to be carried out [11]. Finally, Bergšpica contributed to this Special Issue with a review on aspects related to the occurrence and transmission routes of ESBL producing *Enterobacteriaceae* in the pork production chain. Overall, an increasing trend in the prevalence of presumptive ESBL producing *Escherichia coli* in pigs in the EU has been observed in the last decade. Although the prevalence of ESBL producing *Escherichia coli* is lower in pork meat than in pigs, pork meat can act as a transmission route for this group of critically important AMR bacteria [12].

This Special Issue collects multidisciplinary researches related to the possible emergence, spread and transfer of AMR in the food chain. The contributions here collected constitute a valuable knowledge reservoir for scientists working in the field of AMR.

## References

[1] (2020). The European Union Summary Report on Antimicrobial resistance in zoonotic and indicator bacteria from humans, animals and food in 2017/2018. EFSA J..

[2] Oniciuc E.-A., Likotrafiti E., Alvarez-Molina A., Prieto M., López M., Alvarez-Ordóñez A. (2019). Food processing as a risk factor for antimicrobial resistance spread along the food chain. Curr. Opin. Food Sci..

[3] Rodríguez-López P., Filipello V., Di Ciccio P.A., Pitozzi A., Ghidini S., Scali F., Ianieri A., Zanardi E., Losio M.N., Simon A.C. (2020). Assessment of the Antibiotic Resistance Profile, Genetic Heterogeneity and Biofilm Production of Methicillin-Resistant *Staphylococcus aureus* (MRSA) Isolated from The Italian Swine Production Chain. Foods.

[4] Teixeira P., Fernandes B., Silva A.M., Dias N., Azeredo J. (2020). Evaluation by Flow Cytometry of *Escherichia coli* Viability in Lettuce after Disinfection. Antibiotics.

[5] Bissong M.E.A., Ateba C.N. (2020). Genotypic and Phenotypic Evaluation of Biofilm Production and Antimicrobial Resistance in *Staphylococcus aureus* Isolated from Milk, North West Province, South Africa. Antibiotics.

[6] Peruzy M.F., Capuano F., Proroga Y.T.R., Cristiano D., Carullo M.R., Murru N. (2020). Antimicrobial Susceptibility Testing for Salmonella Serovars Isolated from Food Samples: Five-Year Monitoring (2015–2019). Antibiotics.

[7] Xu X., Biswas S., Gu G., Elbediwi M., Li Y., Yue M. (2020). Characterization of Multidrug Resistance Patterns of Emerging *Salmonella enterica* Serovar Rissen along the Food Chain in China. Antibiotics.

[8] Casagrande-Proietti P.C., Stefanetti V., Musa L., Zicavo A., Dionisi A.M., Bellucci S., La Mensa A., Menchetti L., Branciari R., Ortenzi R. (2020). Genetic Profiles and Antimicrobial Resistance Patterns of *Salmonella* Infantis Strains Isolated in Italy in the Food Chain of Broiler Meat Production. Antibiotics.

[9] Zhou Y., Li X., Yan H. (2020). Genotypic Characteristics and Correlation of Epidemiology of *Staphylococcus aureus* in Healthy Pigs, Diseased Pigs, and Environment. Antibiotics.

[10] Abass A., Adzitey F., Huda N. (2020). *Escherichia coli* of Ready-to-Eat (RTE) Meats Origin Showed Resistance to Antibiotics Used by Farmers. Antibiotics.

[11] Cuccato M., Rubiola S., Giannuzzi D., Grego E., Pregel P., Divari S., Cannizzo F.T. (2021). 16S rRNA Sequencing Analysis of the Gut Microbiota in Broiler Chickens Prophylactically Administered with Antimicrobial Agents. Antibiotics.

[12] Bergšpica I., Kaprou G., Alexa E.A., Prieto M., Alvarez-Ordóñez A. (2020). Extended Spectrum β-Lactamase (ESBL) Producing *Escherichia coli* in Pigs and Pork Meat in the European Union. Antibiotics.

